# Supplementary material for: Organophosphate Flame Retardants and Perfluoroalkyl Substances in Drinking Water Treatment Plants from Korea: Occurrence and Human Exposure
Source: Int J Environ Res Public Health. 2021 Mar 5;18(5):2645. doi: 10.3390/ijerph18052645 (PMC7967649; doi:10.3390/ijerph18052645)
Supplement: Supplementary file 1 [file ijerph-18-02645-s001.pdf]

Supplementary Materials

Organophosphate flame retardants and perfluoroalkyl substances in drinking water treatment plants from Korea: occurrence and human exposure

Wonjin Sim [1], Sol Choi [2], Gyojin Choo [2,3], Mihee Yang [4], Ju-Hyun Park [4], Jeong-Eun Oh [2,*]

[1] Education & Research Center for Infrastructure of Smart Ocean City (i-SOC center), Pusan National University, Busan 46241, Republic of Korea

[2] Department of Civil and Environmental Engineering, Pusan National University, Busan 46241, Republic of Korea

[3] National Fishery Products Quality Management Service, Busan 48943, Republic of Korea

[4] Department of Environmental Infrastructure Research, National Institute of Environmental Research, Ministry of Environment, Incheon 22689, Republic of Korea

***Corresponding author:** Jeong-Eun Oh

Tel.: +82-51-510-3513

E-mail address: jeoh@pusan.ac.kr

Section S1. Chemicals and Materials

TEP, TCEP, TCIPP, TDCIPP, TPhP, TCP, TNBP, TPrP, EHDPP, and DCP were purchased from Accustandard (New Haven, CT, USA). TBOEP and TEHP were obtained from Wellington Laboratories (Guelph, Canada). TIBP was purchased from Toronto Research Chemicals (Ontario, Canada). Deuterated internal standards of OPFR (TCEP-d_{12}, TCIPP-d_{18}, TDCIPP-d_{15}, and TPhP-d_{15}) were purchased from Cambridge Isotope Laboratories (Andover, MA, USA), and phenanthrene-d_{10} (Accustandard) was used as a syringe standard for OPFR. Mass-labelled internal standards for PFAS consist of eight mass-labeled compounds ($^{13}C_2$-PFHxA [MPFHxA], $^{13}C_4$-PFOA [MPFOA], $^{13}C_5$-PFNA [MPFNA], $^{13}C_2$-PFDA [MPFDA], $^{13}C_2$-PFUnDA [MPFUnDA], $^{13}C_2$-PFDoDA [MPFDoDA], $^{18}O_2$-PFHxS [MPFHxS], and $^{13}C_4$-PFOS [MPFOS]), and two syringe standards were $^{13}C_8$-PFOA [M8PFOA] and $^{13}C_8$-PFOS [M8PFOS]. All standards of PFAS were purchased from Wellington Laboratories. Ammonium acetate was obtained from Wako (Osaka, Japan), and DCM was purchased from J.T. Baker (Phillipsburg, NJ, USA). Acetonitrile, water, and MeOH were obtained from Burdick & Jackson (Muskegon, MI, USA).

Table S1. Details of each sample in this study

Region		DWTP	Advanced treatment processes	Capacity (m^3/d)	Water supply type
Upstream	A	1	Ozonation + GAC	400,000	Surface river water
		2	-	64,000	Surface river water
	B	3	Ozonation + GAC	44,000	Surface river water
	C	4	Ozonation + GAC	210,000	Surface river water
		5	Ozonation + GAC	770,000	Surface river water
Midstream	D	6	Ozonation + GAC	22,000	Riverbed water
	E	7	Ozonation + GAC	440,000	Surface river water
		8	Ozonation + GAC	285,000	Surface river water
		9	GAC	11,000	Riverbank filtration
		10	GAC	126,000	Riverbank filtration
Downstream	F	11	Ozonation + GAC	270,000	Surface river water
		12	Ozonation + GAC		
	G	13	Ozonation + GAC	1st: 450,000<br>2nd: 825,000	Surface river water
		14	Ozonation + GAC	74,200	Surface water
		15	Ozonation + GAC		
	H	16	Ozonation + GAC	1st: 450,000<br>2nd: 825,000	Surface water
	I	17	Ozonation + GAC	1,725,000	Surface water
		18	Ozonation + GAC	840,000	Surface water

Table S2. Conditions of instrumental analysis for OPFR

**Parameter**	**Conditions**
Column	DB-5MS UI (15 m × 0.25 mm I.D., 0.10 μm film thickness)
Carrier gas	He (99.9999%) at 1.5 mL/min
Injection temp.	300 ℃
Injection mode	Splitless
Column temp.	50 ℃ (3 min) – 15 ℃/min – 300 ℃ (1 min)
Interface temp.	280 ℃
Source temp.	300 ℃
Ionization voltage	70 eV
Ionization mode	EI

Table S3. SRM conditions of OPFR

Compound	Precursor ion (m/z)	Product ions (m/z)		Collision energy (eV)	
		Quantitative	Confirm	Quantitative	Confirm
TEP	99	81	63	22	42
TPrP	141	99	81	7	35
TIBP	99	81	63	22	42
TNBP	99	81	63	22	42
TCEP	249	125	99	11	41
TCIPP	125	99	63	17	40
TDCIPP	99	81	63	22	42
TBOEP	125	99	63	17	40
TPhP	326	169	77	43	44
EHDPP	251	77.1	152.1	40	39
TEHP	99	81	63	22	42
DCP	340	165	183	39	39
TCP	368	165	91	40	44
TCEP-d_{12}	261	103	67	25	40
TCIPP-d_{18}	131	83	63	18	43
TDCIPP-d_{15}	103	83	63	24	40
TPhP-d_{15}	341	243	180	10	20
Phenanthrene-d_{10}	188	160	136	32	40

Table S4. Conditions of instrumental analysis for PFAS

Parameter	Conditions						
Column	ZORBAX Eclipse XDB-C18 (4.6 × 150 mm, 3.5 μm)						
Mobile phase	A: Methanol						
	B: 2mM Ammonium acetate in water						
Gradient	Time (min)	0	10	13	29	31	45
	Solvent B (%)	70	70	30	10	0	0
Flow rate	300 μL/min						
Injection volume	10 μL						
Column temp.	25 ℃						
Ionization mode	Negative ion electrospray						
Gas temp.	300 ℃ (Sheath: 350 ℃)						
Gas flow	8 L/min (Sheath: 12 L/min)						
Capillary voltage	4000 V						
Nebulizer	40 psi						

Table S5. SRM conditions of PFAS

Compound	Precursor ion (m/z)	Product ions (m/z)		Fragment energy (V)	Collision energy (eV)	
		Quantitative	Confirm		Quantitative	Confirm
PFPeA	263	263	219	60	2	2
PFHxA	313	269	119	65	3	10
PFHpA	363	319	169	70	2	13
PFOA	413	369	169	70	1	9
PFNA	463	419	219	80	4	6
PFDA	513	469	219	85	4	14
PFUnDA	563	519	269	80	2	12
PFDoDA	613	569	319	90	1	14
PFTrDA	663	619	219	90	6	9
PFTeDA	713	669	419	100	11	20
L-PFBS	299	99	80	120	28	28
L-PFPeS	349	99	80	150	30	30
L-PFHxS	399	99	80	114	38	40
B-PFHxS	399	99	80	114	38	40
L-PFHpS	449	99	80	140	40	40
L-PFOS	499	99	80	131	49	50
B-PFOS	499	99	80	131	49	50
L-PFNS	549	99	80	180	48	56
L-PFDS	599	99	80	180	73	71
N-MeFOSAA	570	419	469	152	16	20
N-EtFOSAA	584	419	526	114	18	12
4:2FTS	327	307	80	120	20	30
6:2FTS	427	407	80	130	20	42
8:2FTS	527	507	80	165	26	30
ADONA	377	251	85	80	4	35
GenX	329	285	169	60	0	8
9Cl-PF3ONS	531	351	-	145	24	-
MPFHxA	315	315	270	78	1	1
MPFOA	417	372	172	75	2	15
MPFNA	468	423	223	97	1	16
MPFDA	515	515	470	96	1	3

MPFUndA	565	565	520	113	1	3
MPFDoA	615	570	270	111	2	20
MPFHxS	403	103	84	139	43	65
MPFOS	503	99	80	150	50	53
M8PFOA	421	376	172	50	4	4
M8PFOS	506	99	80	170	72	72

Table S6. Detailed concentrations of OPFR and PFAS in DWTPs (unit: ng/L)

Compound	MDL	Raw water (n = 36)					Treated water (n = 36)				
		DF (%)	Min	Max	Mean	Median	DF (%)	Min	Max	Mean	Median
TEP	0.78	97	ND	33.7	10.0	7.90	78	ND	24.0	3.41	2.93
TPrP	1.10	0	-	-	-	-	0	-	-	-	-
TIBP	0.60	81	ND	9.62	3.64	3.65	86	0.00	14.7	2.63	2.39
TNBP	0.23	97	ND	9.71	4.46	4.05	100	0.76	7.03	2.44	2.26
TCEP	0.28	100	8.24	143	30.5	26.9	100	7.13	74.9	17.3	11.8
TCIPP	0.26	100	10.0	55.1	34.0	30.8	100	4.57	44.6	17.9	16.2
TDCIPP	0.51	42	ND	2.62	0.65	ND	11	ND	1.92	0.15	ND
TBOEP	0.77	100	5.32	25.0	13.3	12.1	100	4.48	19.3	8.32	7.52
TPhP	0.30	58	ND	5.73	2.37	2.29	67	ND	6.38	1.73	2.34
EHDPP	0.29	97	ND	3.98	3.13	3.14	72	ND	2.35	0.89	0.63
TEHP	0.26	50	ND	1.70	0.58	0.45	53	ND	0.85	0.27	0.46
DCP	0.45	0	-	-	-	-	0	-	-	-	-
TCP	0.30	0	-	-	-	-	0	-	-	-	-
$\sum_{13}$OPFR		100	37.7	231	103	98.1	100	29.5	122	55.1	47.5
PFPeA	0.30	64	ND	12.3	3.62	3.96	67	ND	12.4	4.68	4.89
PFHxA	0.43	100	1.75	19.6	8.18	8.03	100	1.89	20.6	9.54	9.52
PFHpA	0.32	100	0.81	6.09	3.20	3.26	94	ND	8.26	3.64	3.83
PFOA	0.39	97	ND	21.1	9.23	8.82	100	1.52	27.1	10.3	9.94
PFNA	0.38	100	0.48	6.12	1.63	1.34	69	ND	4.99	1.39	1.37
PFDA	0.50	36	ND	22.0	1.14	ND	44	ND	2.66	0.64	ND
PFUnDA	0.43	11	ND	1.66	0.08	ND	0	-	-	-	-
PFDoDA	0.39	0	-	-	-	-	0	-	-	-	-
PFTrDA	0.42	0	-	-	-	-	0	-	-	-	-
PFTeDA	0.80	0	-	-	-	-	0	-	-	-	-
$\sum_{10}$PFCAs		100	3.33	76.1	27.1	25.9	100	3.58	68.4	30.2	28.8
L-PFBS	0.31	97	ND	7.86	2.25	1.78	94	ND	7.73	2.40	1.70
L-PFPeS	0.33	56	ND	0.78	0.32	0.49	6	ND	0.99	0.05	ND
L-PFHxS	0.21	89	ND	42.2	5.66	1.46	92	ND	37.7	13.3	8.69
B-PFHxS	0.48	33	ND	7.93	0.95	ND	78	ND	8.25	2.27	1.42
L-PFHpS	0.37	0	-	-	-	-	0	-	-	-	-
L-PFOS	0.46	94	ND	16.8	1.56	0.96	67	ND	3.28	1.04	1.09
B-PFOS	0.20	83	ND	6.67	0.98	0.91	47	ND	2.45	0.56	ND
L-PFNS	0.39	11	ND	0.44	0.05	ND	0	-	-	-	-
L-PFDS	0.69	0	-	-	-	-	0	-	-	-	-
$\sum_{9}$PFSAs		100	0.51	77.4	11.8	6.16	100	0.73	53.6	19.6	14.2
N-MeFOSAA	0.64	0	-	-	-	-	0	-	-	-	-
N-EtFOSAA	0.54	0	-	-	-	-	8	ND	0.62	0.05	ND
4:2FTS	1.09	0	-	-	-	-	0	-	-	-	-
6:2FTS	0.99	11	ND	5.67	0.38	ND	19	ND	2.23	0.29	ND
8:2FTS	1.03	0	-	-	-	-	0	-	-	-	-
$\sum_{5}$Precursors		11	ND	5.67	0.38	ND	22	ND	2.44	0.34	ND
ADONA	0.35	0	-	-	-	-	0	-	-	-	-
GenX	0.58	0	-	-	-	-	0	-	-	-	-
9Cl-PF3ONS	0.57	0	-	-	-	-	0	-	-	-	-
$\sum_{3}$Alternatives		0	-	-	-	-	0	-	-	-	-
$\sum_{27}$PFAS		100	4.15	154	39.2	32.0	100	4.74	116	50.2	42.2

MDL: method detection limit, DF: detection frequency, ND: not detected (< MDL)

Table S7. Concentrations of OPFR and PFAS in 2017 and 2018 (unit: ng/L)

Year	Compound	Raw water				Treated water			
		Min	Max	Mean	Median	Min	Max	Mean	Median
2017	TEP	ND	10.3	5.02	5.01	ND	9.62	4.69	4.61
	TNBP	1.81	11.8	4.89	4.03	0.47	4.51	1.81	1.67
	TCEP	9.89	68.9	32.5	28.0	8.13	29.0	18.4	18.6
	TCIPP	7.09	109	45.5	44.8	7.05	44.7	24.0	21.8
	TDCIPP	ND	7.20	3.83	4.74	ND	6.85	3.03	2.90
	TBOEP	7.56	52.8	22.8	22.6	1.81	35.3	9.61	5.84
	TPhP	2.69	54.2	9.33	6.32	1.33	17.6	4.28	3.75
	TEHP	ND	6.44	3.07	3.05	ND	6.19	3.06	3.05
	TCP	ND	3.85	0.19	ND	ND	1.35	0.10	ND
	∑OPFR	39.1	245	127	113	30.8	123	69.1	62.6
2017	PFPeA	2.6	16.5	7..4	7.4	1.5	17.9	6.0	5.6
	PFHxA	1.4	32.9	9.8	8.3	1.5	33.7	9.3	7.7
	PFHpA	0.7	12	4.0	3.5	0.6	12.8	3.6	2.5
	PFOA	1.9	65.2	15.9	11.4	1.5	64.9	11.9	7.6
	PFNA	0.5	5.1	2.4	2.4	0.3	4.8	1.5	1.2
	PFDA	ND	3.5	0.9	0.7	ND	2.8	0.4	0.3
	PFUnDA	ND	0.3	0.1	ND	ND	0.13	0.01	ND
	PFBS	ND	10.7	2.7	1.91	ND	9.2	2.2	1.5
	PFHxS	0.5	599.6	106	42.9	0.5	454.2	69.6	18.0
	PFOS	0.5	4.6	2.0	1.9	ND	3.9	0.9	0.7
	∑PFAS	8.99	644.6	151.1	93.8	6.3	493.1	105.5	65.1
2018	PFPeA	-	-	-	-	ND	7	1.9	1.5
	PFHxA	-	-	-	-	3	22	12.6	14.0
	PFHpA	-	-	-	-	2	10	5.6	6.0
	PFOA	-	-	-	-	1	43	16.2	14.5
	PFNA	-	-	-	-	0	7	2.7	2.0
	PFDA	-	-	-	-	2	6	3.6	3.0
	PFHxS	-	-	-	-	ND	126	52.2	48.5
	PFOS	-	-	-	-	2	10	4.5	3.0
	∑PFAS	-	-	-	-	10	173	99.3	106

Table S8. Estimated daily intakes of OPFR and PFAS by the Monte Carlo simulation

Compound	Age	R/BW (mL/kg/d)		Estimated daily intake (ng/kg/d)					
				Mean	Range		Selected percentile		
		Mean	SD		Min	Max	P5	P50	P95
$\sum_{13}$OPFR	Adult	15.93	10.92	9.7×10^{-1}	3.0×10^{-5}	7.1×10^{0}	1.5×10^{-1}	8.5×10^{-1}	2.2×10^{0}
	13 - 18	16.09	9.99	9.5×10^{-1}	1.1×10^{-4}	6.1×10^{0}	1.6×10^{-1}	8.3×10^{-1}	2.1×10^{0}
	7 - 12	23.06	15.17	1.4×10^{0}	2.0×10^{-5}	9.1×10^{0}	2.1×10^{-1}	1.2×10^{0}	3.0×10^{0}
	3 - 6	32.42	19.04	1.9×10^{0}	3.2×10^{-4}	1.0×10^{1}	3.5×10^{-1}	1.7×10^{0}	4.2×10^{0}
	1 - 2	36.60	25.30	2.2×10^{0}	1.7×10^{-3}	1.7×10^{1}	3.2×10^{-1}	1.9×10^{0}	4.9×10^{0}
$\sum_{27}$PFAS	Adult	15.93	10.92	8.9×10^{-1}	3.0×10^{-5}	7.8×10^{0}	6.5×10^{-2}	6.6×10^{-1}	2.5×10^{0}
	13 - 18	16.09	9.99	8.6×10^{-1}	1.0×10^{-5}	8.2×10^{0}	6.5×10^{-2}	6.5×10^{-1}	2.4×10^{0}
	7 - 12	23.06	15.17	1.3×10^{0}	1.4×10^{-4}	1.2×10^{1}	9.1×10^{-2}	9.3×10^{-1}	3.6×10^{0}
	3 - 6	32.42	19.04	1.7×10^{0}	6.0×10^{-5}	1.6×10^{1}	1.4×10^{-1}	1.3×10^{0}	4.7×10^{0}
	1 - 2	36.60	25.30	2.0×10^{0}	2.8×10^{-4}	1.8×10^{1}	1.5×10^{-1}	1.5×10^{0}	5.7×10^{0}
TNBP	Adult	15.93	10.92	4.3×10^{-2}	0.0×10^{0}	2.9×10^{-1}	7.0×10^{-3}	3.8×10^{-2}	9.4×10^{-2}
	13 - 18	16.09	9.99	4.2×10^{-2}	1.0×10^{-5}	2.4×10^{-1}	7.0×10^{-3}	3.8×10^{-2}	9.1×10^{-2}
	7 - 12	23.06	15.17	6.1×10^{-2}	3.0×10^{-5}	3.5×10^{-1}	1.0×10^{-2}	5.5×10^{-2}	1.4×10^{-1}
	3 - 6	32.42	19.04	8.3×10^{-2}	1.0×10^{-5}	5.5×10^{-1}	1.6×10^{-2}	7.5×10^{-2}	1.8×10^{-1}
	1 - 2	36.60	25.30	9.9×10^{-2}	0.0×10^{0}	5.7×10^{-1}	1.5×10^{-2}	8.7×10^{-2}	2.2×10^{-1}
TCEP	Adult	15.93	10.92	3.0×10^{-1}	1.0×10^{-5}	5.4×10^{0}	3.5×10^{-2}	2.3×10^{-1}	7.8×10^{-1}
	13 - 18	16.09	9.99	2.9×10^{-1}	4.0×10^{-5}	4.0×10^{0}	3.8×10^{-2}	2.2×10^{-1}	7.7×10^{-1}
	7 - 12	23.06	15.17	4.2×10^{-1}	1.8×10^{-4}	6.9×10^{0}	5.0×10^{-2}	3.2×10^{-1}	1.1×10^{0}
	3 - 6	32.42	19.04	5.7×10^{-1}	3.0×10^{-5}	7.5×10^{0}	7.5×10^{-2}	4.4×10^{-1}	1.5×10^{0}
	1 - 2	36.60	25.30	6.8×10^{-1}	1.0×10^{-5}	1.2×10^{0}	8.1×10^{-2}	5.2×10^{-1}	1.8×10^{0}
TCIPP	Adult	15.93	10.92	3.2×10^{-1}	1.0×10^{-5}	2.0×10^{0}	3.8×10^{-2}	2.5×10^{-1}	8.4×10^{-1}
	13 - 18	16.09	9.99	3.1×10^{-1}	1.0×10^{-5}	1.9×10^{0}	4.0×10^{-2}	2.4×10^{-1}	7.9×10^{-1}
	7 - 12	23.06	15.17	4.5×10^{-1}	5.0×10^{-5}	2.8×10^{0}	5.3×10^{-2}	3.5×10^{-1}	1.2×10^{0}
	3 - 6	32.42	19.04	6.1×10^{-1}	4.0×10^{-5}	4.0×10^{0}	8.4×10^{-2}	4.9×10^{-1}	1.6×10^{0}
	1 - 2	36.60	25.30	7.3×10^{-1}	1.4×10^{-4}	4.5×10^{0}	8.6×10^{-2}	5.6×10^{-1}	1.9×10^{0}
TBOEP	Adult	15.93	10.92	1.5×10^{-1}	1.0×10^{-5}	8.4×10^{-1}	2.3×10^{-2}	1.3×10^{-1}	3.3×10^{-1}
	13 - 18	16.09	9.99	1.4×10^{-1}	0.0×10^{0}	8.7×10^{-1}	2.5×10^{-2}	1.3×10^{-1}	3.1×10^{-1}
	7 - 12	23.06	15.17	2.1×10^{-1}	9.0×10^{-5}	1.3×10^{0}	3.2×10^{-2}	1.8×10^{-1}	4.6×10^{-1}
	3 - 6	32.42	19.04	2.8×10^{-1}	2.0×10^{-5}	1.5×10^{0}	5.2×10^{-2}	2.5×10^{-1}	6.1×10^{-1}
	1 - 2	36.60	25.30	3.4×10^{-1}	8.0×10^{-5}	1.9×10^{0}	5.2×10^{-2}	3.0×10^{-1}	7.5×10^{-1}
TPhP	Adult	15.93	10.92	3.8×10^{-2}	0.0×10^{0}	2.9×10^{-1}	1.0×10^{-3}	2.7×10^{-2}	1.1×10^{-1}
	13 - 18	16.09	9.99	3.7×10^{-2}	0.0×10^{0}	2.7×10^{-1}	1.0×10^{-3}	2.6×10^{-2}	1.1×10^{-1}
	7 - 12	23.06	15.17	5.4×10^{-2}	0.0×10^{0}	4.1×10^{-1}	2.0×10^{-3}	3.8×10^{-2}	1.6×10^{-1}
	3 - 6	32.42	19.04	7.3×10^{-2}	0.0×10^{0}	5.7×10^{-1}	3.0×10^{-3}	5.3×10^{-2}	2.1×10^{-1}
	1 - 2	36.60	25.30	8.7×10^{-2}	0.0×10^{0}	7.0×10^{-1}	3.0×10^{-3}	6.0×10^{-2}	2.6×10^{-1}
PFOA	Adult	15.93	10.92	1.8×10^{-1}	1.0×10^{-5}	1.5×10^{0}	1.4×10^{-2}	1.4×10^{-1}	5.1×10^{-1}
	13 - 18	16.09	9.99	1.8×10^{-1}	0.0×10^{0}	1.6×10^{0}	1.5×10^{-2}	1.4×10^{-1}	4.8×10^{-1}
	7 - 12	23.06	15.17	2.6×10^{-1}	3.0×10^{-5}	2.4×10^{0}	2.0×10^{-2}	1.9×10^{-1}	7.3×10^{-1}
	3 - 6	32.42	19.04	3.5×10^{-1}	2.0×10^{-5}	3.1×10^{0}	3.1×10^{-2}	2.7×10^{-1}	9.6×10^{-1}
	1 - 2	36.60	25.30	4.2×10^{-1}	6.0×10^{-5}	3.7×10^{0}	3.3×10^{-2}	3.1×10^{-1}	1.2×10^{0}
PFOS (total)	Adult	15.93	10.92	3.3×10^{-2}	0.0×10^{-2}	2.8×10^{-1}	2.0×10^{-3}	2.4×10^{-2}	9.5×10^{-2}
	13 - 18	16.09	9.99	3.3×10^{-2}	0.0×10^{-2}	2.8×10^{-1}	2.0×10^{-3}	2.4×10^{-2}	9.2×10^{-2}
	7 - 12	23.06	15.17	4.8×10^{-2}	0.0×10^{-2}	3.2×10^{-1}	3.0×10^{-3}	3.5×10^{-2}	1.4×10^{-1}
	3 - 6	32.42	19.04	6.6×10^{-2}	0.0×10^{-2}	4.6×10^{-1}	4.0×10^{-3}	4.9×10^{-2}	1.9×10^{-1}
	1 - 2	36.60	25.30	7.7×10^{-2}	0.0×10^{-2}	6.4×10^{-1}	4.0×10^{-3}	5.6×10^{-2}	2.2×10^{-1}

R/BW: daily consumption rate of drinking water per body weight, SD: standard deviation, P5: 5th percentile, P50: 50th percentile, P95: 95th percentile

Table S9. Potential non-cancer risks of OPFR and PFAS by the Monte Carlo simulation

Compound	RfD or TDI (ng/kg/d)	Age	Hazard quotient (HQ)					
			Mean	Range		Selected percentile		
				Min	Max	P5	P50	P95
TNBP	2400	Adult	1.8×10^{-5}	0.0×10^{0}	1.2×10^{-4}	2.9×10^{-6}	1.6×10^{-5}	3.9×10^{-5}
		13 - 18	1.8×10^{-5}	4.2×10^{-9}	1.0×10^{-4}	2.9×10^{-6}	1.6×10^{-5}	3.8×10^{-5}
		7 - 12	2.5×10^{-5}	1.3×10^{-8}	1.5×10^{-4}	4.2×10^{-6}	2.3×10^{-5}	5.6×10^{-5}
		3 - 6	3.5×10^{-5}	4.2×10^{-9}	2.3×10^{-4}	6.7×10^{-6}	3.1×10^{-5}	7.5×10^{-5}
		1 - 2	4.1×10^{-5}	0.0×10^{0}	2.4×10^{-4}	6.3×10^{-6}	3.6×10^{-5}	9.2×10^{-5}
TCEP	2200	Adult	1.3×10^{-4}	4.5×10^{-9}	2.5×10^{-3}	1.6×10^{-5}	1.0×10^{-4}	3.6×10^{-4}
		13 - 18	1.3×10^{-4}	1.8×10^{-8}	1.8×10^{-3}	1.7×10^{-5}	1.0×10^{-4}	3.5×10^{-4}
		7 - 12	1.9×10^{-4}	8.2×10^{-8}	3.1×10^{-3}	2.3×10^{-5}	1.5×10^{-4}	5.1×10^{-4}
		3 - 6	2.6×10^{-4}	1.4×10^{-8}	3.4×10^{-3}	3.4×10^{-5}	2.0×10^{-4}	6.8×10^{-4}
		1 - 2	3.1×10^{-4}	4.5×10^{-9}	5.4×10^{-3}	3.7×10^{-5}	2.3×10^{-4}	8.3×10^{-4}
TCIPP	8000	Adult	4.0×10^{-5}	1.3×10^{-9}	2.5×10^{-4}	4.8×10^{-6}	3.1×10^{-5}	1.0×10^{-4}
		13 - 18	3.9×10^{-5}	1.3×10^{-9}	2.4×10^{-4}	5.0×10^{-6}	3.0×10^{-5}	9.8×10^{-5}
		7 - 12	5.6×10^{-5}	6.3×10^{-9}	3.6×10^{-4}	6.6×10^{-6}	4.4×10^{-5}	1.5×10^{-4}
		3 - 6	7.7×10^{-5}	5.0×10^{-9}	5.0×10^{-4}	1.1×10^{-5}	6.1×10^{-5}	2.0×10^{-4}
		1 - 2	9.1×10^{-5}	1.8×10^{-8}	5.6×10^{-4}	1.1×10^{-5}	7.0×10^{-5}	2.4×10^{-4}
TBOEP	1500	Adult	9.7×10^{-5}	6.7×10^{-9}	5.6×10^{-4}	1.5×10^{-5}	8.7×10^{-5}	2.2×10^{-4}
		13 - 18	9.5×10^{-5}	0.0×10^{0}	5.8×10^{-4}	1.7×10^{-5}	8.5×10^{-5}	2.0×10^{-4}
		7 - 12	1.4×10^{-4}	6.0×10^{-8}	8.5×10^{-4}	2.1×10^{-5}	1.2×10^{-4}	3.1×10^{-4}
		3 - 6	1.9×10^{-4}	1.3×10^{-8}	1.0×10^{-3}	3.5×10^{-5}	1.7×10^{-4}	4.1×10^{-4}
		1 - 2	2.2×10^{-4}	5.3×10^{-8}	1.3×10^{-3}	3.5×10^{-5}	2.0×10^{-4}	5.0×10^{-4}
TPhP	7000	Adult	5.4×10^{-6}	0.0×10^{0}	4.1×10^{-5}	1.4×10^{-7}	3.9×10^{-6}	1.6×10^{-5}
		13 - 18	5.3×10^{-6}	0.0×10^{0}	3.8×10^{-5}	1.4×10^{-7}	3.7×10^{-6}	1.5×10^{-5}
		7 - 12	7.7×10^{-6}	0.0×10^{0}	5.9×10^{-5}	2.9×10^{-7}	5.4×10^{-6}	2.3×10^{-5}
		3 - 6	1.0×10^{-5}	0.0×10^{0}	8.1×10^{-5}	4.3×10^{-7}	7.6×10^{-6}	3.0×10^{-5}
		1 - 2	1.2×10^{-5}	0.0×10^{0}	1.0×10^{-4}	4.3×10^{-7}	8.6×10^{-6}	3.7×10^{-5}
PFOA	1500	Adult	1.2×10^{-4}	6.7×10^{-9}	1.0×10^{-3}	9.3×10^{-6}	9.3×10^{-5}	3.4×10^{-4}
		13 - 18	1.2×10^{-4}	0.0×10^{0}	1.1×10^{-3}	1.0×10^{-5}	9.1×10^{-5}	3.2×10^{-4}
		7 - 12	1.7×10^{-4}	2.0×10^{-8}	1.6×10^{-3}	1.3×10^{-5}	1.3×10^{-4}	4.8×10^{-4}
		3 - 6	2.4×10^{-4}	1.3×10^{-8}	2.1×10^{-3}	2.1×10^{-5}	1.8×10^{-4}	6.4×10^{-4}
		1 - 2	2.8×10^{-4}	4.0×10^{-8}	2.4×10^{-3}	2.2×10^{-5}	2.1×10^{-4}	7.8×10^{-4}
PFOS (total)	150	Adult	2.2×10^{-4}	0.0×10^{0}	1.9×10^{-3}	1.3×10^{-5}	1.6×10^{-4}	6.3×10^{-4}
		13 - 18	2.2×10^{-4}	0.0×10^{0}	1.9×10^{-3}	1.3×10^{-5}	1.6×10^{-4}	6.1×10^{-4}
		7 - 12	3.2×10^{-4}	0.0×10^{0}	2.2×10^{-3}	2.0×10^{-5}	2.3×10^{-4}	9.1×10^{-4}
		3 - 6	4.4×10^{-4}	0.0×10^{0}	3.1×10^{-3}	2.7×10^{-5}	3.3×10^{-4}	1.2×10^{-3}
		1 - 2	5.1×10^{-4}	0.0×10^{0}	4.3×10^{-3}	2.7×10^{-5}	3.7×10^{-4}	1.5×10^{-3}
Hazard index (HI)	-	Adult	6.4×10^{-4}	1.9×10^{-8}	6.3×10^{-3}	6.2×10^{-5}	4.9×10^{-4}	1.7×10^{-3}
		13 - 18	6.3×10^{-4}	2.4×10^{-8}	5.7×10^{-3}	6.5×10^{-5}	4.9×10^{-4}	1.6×10^{-3}
		7 - 12	9.1×10^{-4}	1.8×10^{-7}	8.3×10^{-3}	8.8×10^{-5}	7.0×10^{-4}	2.4×10^{-3}
		3 - 6	1.2×10^{-3}	4.9×10^{-8}	1.0×10^{-2}	1.3×10^{-4}	9.8×10^{-4}	3.3×10^{-3}
		1 – 2	1.5×10^{-3}	1.2×10^{-7}	1.4×10^{-2}	1.4×10^{-4}	1.1×10^{-3}	3.9×10^{-3}

RfD: oral reference dose, TDI: tolerable daily intake, P5: 5th percentile, P50: 50th percentile, P95: 95th percentile

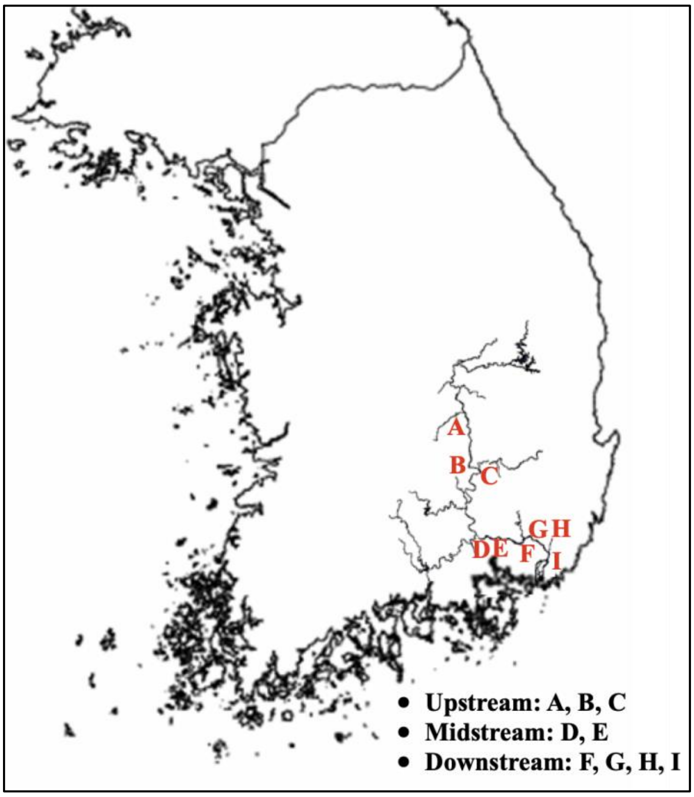

Figure S1. Locations of water supplies in the Nakdong River.

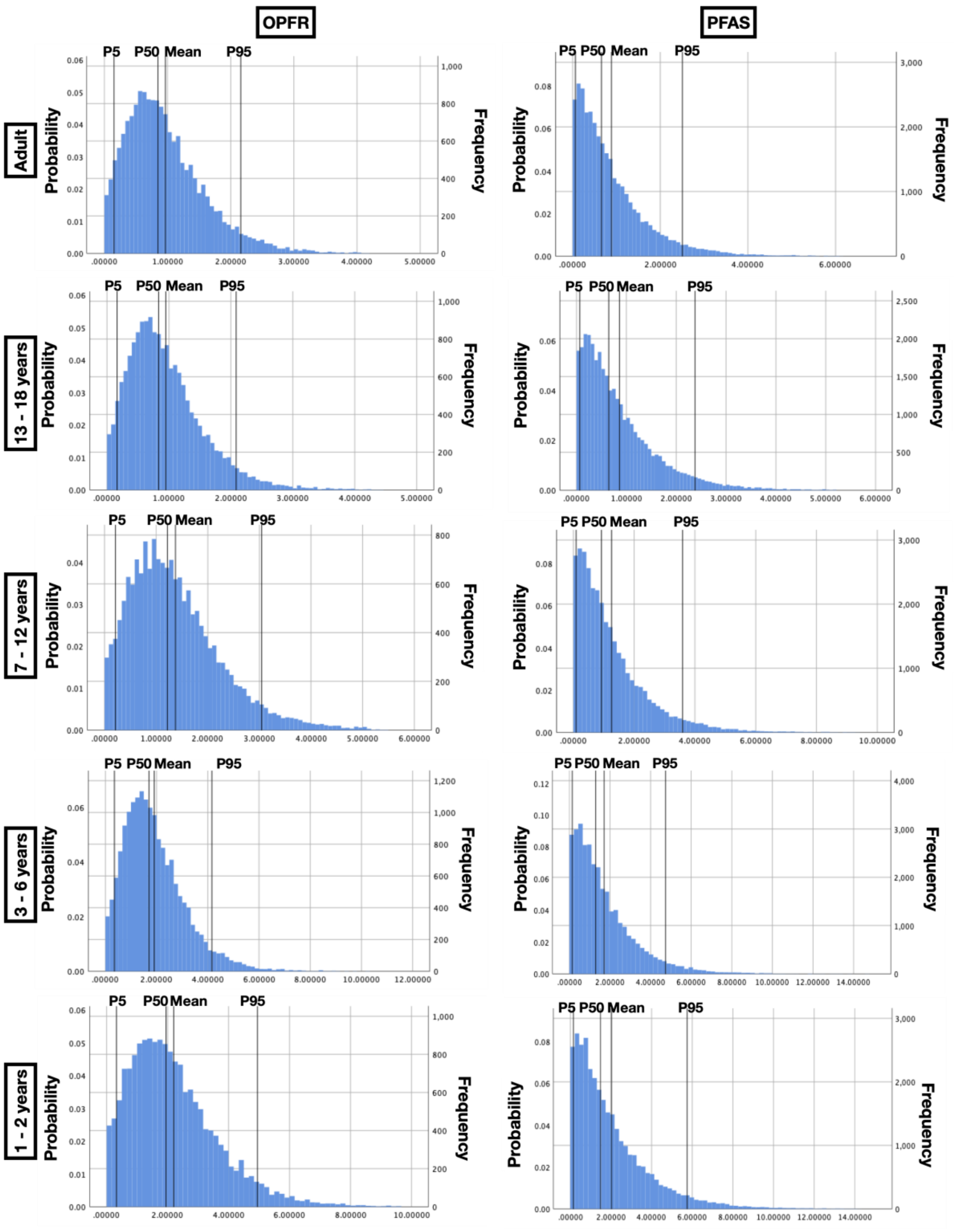

Figure S2. Frequency charts of daily intake by the Monte Carlo simulation.
